# Sugarcane cultivation reduces charcoal C and Al/Fe-bound OC in selected Ferralsols

**DOI:** 10.1007/s10661-025-13924-8

**Published:** 2025-03-31

**Authors:** Nontokozo Pertunia Mkhonza, Pardon Muchaonyerwa

**Affiliations:** Soil Science Discipline, School of Agricultural, Earth and Environmental Sciences, University of Kwazulu-Natal, Private Bag X01, Scottsville, Pietermaritzburg, 3201 South Africa

**Keywords:** Acidic soils, Carbon stabilisation, Organo-mineral complexes, Soil carbon sequestration

## Abstract

Long-term sugarcane cultivation, with pre-harvest burning, may add recalcitrant charcoal carbon (C) to soil organic carbon (OC) in Ferralsols, whereas aluminium/iron-organic matter (Al/Fe-OM) complexes may dominate the mineral-associated OC in these soils. Therefore, the objective of this study was to examine the effect of sugarcane cultivation relative to wattle forest on soil OC in charcoal C form and Al/Fe-OM complexes on two selected Ferralsols. Total C, charcoal C, and Al/Fe-OC were analysed in samples collected from the two sites under forest and sugarcane cultivation to a depth of 100 cm, and the data were subjected to a two-way analysis of variance. At both sites, sugarcane cultivation reduced charcoal C and Al/Fe-bound OC when compared to forest. The Al/Fe-bound OC in sugarcane soils accounted for 48.7 and 72.2% of the total OC at Eston and Wartburg sites, respectively. In forest soils, the Al/Fe-bound OC accounted for 45.6 and 44.4% of the total OC at Eston and Wartburg, respectively. Charcoal C accounted for 8.42 and 4.07% of the total OC in sugarcane soils, at Eston and Wartburg, respectively. Overall, charcoal C concentration decreased with increase in soil depth, while its stocks only decreased with depth in soil under sugarcane. The Al/Fe-bound OC decreased with an increase in soil depth for both land uses, while Alp and Alp + Fep concentrations were not affected by sampling depth. These findings demonstrate that OC in Al/Fe-OC complexes and charcoal C contribute to the high OC concentrations, with Al/Fe-bound OC fraction being the primary mechanism of OC stabilisation in these Ferralsols, while sugarcane cultivation reduces these concentrations.

## Introduction

The capacity of soils to retain significant quantities of organic carbon (OC) has sparked increased interest in investigating the underlying mechanisms of OC stabilisation in different soil environments (Sun et al., 2019). Comprehending these mechanisms is crucial because they have a significant influence on climate change mitigation, soil fertility, and food security (Wang et al., 2010; Wei et al., 2021). The formation of organo-mineral complexes and physical protection in water-stable aggregates are the primary mechanisms of OC stabilisation that have been documented in the literature. Lawrence et al. (2015) found that the presence of aluminium/ iron-organic matter (Al/Fe-OM) complexes increases the organic matter accumulation and reduces its susceptibility to microbial degradation. The same authors reported a strong association between the concentration of OC and the presence of Fe and Al complexes with organic matter in volcanic soils. This finding emphasises the importance of these complexes in the stabilisation of OC in volcanic soils (Shindo & Inoue, 2024). In addition to mineral-associated OC, which includes of organo-mineral complexes, charcoal C is another stable component of OC that has received less attention in the literature. A study conducted by Briggs et al. (2012) demonstrated that charcoal C constitutes 5–30% of the total OC concentration in soil.

Historically, researchers have recognised black C, also known as charcoal C, for its high resistance to microbial attack and degradation (Kopecky et al., 2021; Lehmann et al., 2007). Additionally, it is regarded as an inactive reservoir of C in soils, serving as a vital repository for global C (Briggs et al., 2012; Kopecky et al., 2021). Charcoal C exhibits greater resistance to breakdown due to its greater C: N ratio (Schmidt & Noack, 2000). Moreover, researchers have observed that the hydrophobic substances, such as polycyclic aromatic hydrocarbons produced during combustion, improve the stability of OC (Doerr et al., 1998; Spaccini et al., 2006). Several researchers have investigated the levels of charcoal C in different types of land use, such as forests, pastures, and croplands, which are produced during the burning of aboveground residues. The studies conducted by Eckmeier et al. (2007), Hirsch et al. (2017), Sedano et al. (2016), Wang et al. (2017), and Mastrolonardo et al. (2018) provide insights into this matter. While the act of burning crop residues to convert natural forests into cropland can increase the concentration of charcoal C, there is limited knowledge regarding its stability in cropped soil profiles. The charcoal C in these soils could be produced from the pre-harvest burning of residues during harvesting, which then accumulates in the soil profile as charcoal C.

Most studies focus on the topsoil layer, which greatly affects crop productivity while overlooking the substantial role of deeper soil layers on OC sequestration. Incorporating OC into deeper layers is crucial for effectively sequestering OC. This is because deeper layers experience less disturbance and have less oxygen availability, which restricts microbial activities and leads to decreased breakdown. Organic C has the potential to be stored in the subsoil in the form of organo-mineral complexes and particulate organic matter (POM-C), which may include charcoal C. Jobbagy and Jackson (2000) found that the concentration of OC in the top 20-cm depth of soil had 33, 42, and 50% of the total OC in 100-cm-depth soil profiles under shrubland, grassland, and forest, respectively. This suggests that the deeper layers of the soil store a significant amount of the OC. It is crucial to have a comprehensive understanding of how site characteristics such as climate, soil conditions, and land use affect the concentration of stable pools of OC, including charcoal C and OC in Al/Fe-OM complexes. This understanding is particularly important in Ferralsols, which are among the most productive soils in South Africa.

The Ferralsols that are locally known as humic soils are highly weathered and acidic, with high C content (> 1.8%) throughout the depth of the top soil horizon (Fey, 2010; Soil Classification Working Group, 2018) and are characterised by low base status and are well drained (Fey, 2010). In a study conducted on selected humic soils of South Africa, Malepfane et al. (2022) found that the thick (> 45-cm depth) and thin (< 45-cm depth) humic A horizons can store up to 70 and 50% of the total C in the soil profile (100-cm depth), respectively. They also observed that, although cultivation led to a decrease in C concentrations in the topsoil, it did not have a significant impact on the overall C stocks in the soil profile compared to uncultivated areas. According to Malepfane et al. (2023), POM-C accounted for over 60% of the total C content in the upper 30-cm depth of soil. This indicates that this OC, which is potentially easily decomposed by microorganisms, is protected from microbial degradation. Nevertheless, the specific process by which OC remains stable in these soils up to deeper depths is still not understood. Malepfane et al. (2022) attributed the greater OC content in humic soils to the lower pH levels (pH < 5.00) and lower concentrations of exchangeable bases (< 4 cmol ( +) per kg clay for every 1% OC present) (Soil Classification Working Group, 2018), which may have hindered microbial activity. Furthermore, Malepfane et al. (2023) found that POM-C was the predominant OC in both the macro- and micro-aggregate size fractions. Additionally, some of the POM-C could be present in the form of charcoal C, while the OC associated with minerals, such as silt and clay, may be predominantly composed of Al/Fe-organic matter complexes.

The recalcitrant nature of charcoal C and the greater acidity in humic soils may lead to the build-up of charcoal C and the formation of Al/Fe-OM complexes. The Alp and Fep are poorly crystallised minerals and have a greater surface area, which affects C in soils (Fang et al., 2019). Verde et al. (2005) reported that Andosols showed a significantly greater concentration of Alp in uncultivated forestry (10.2 g kg^−1^) compared to cultivated agricultural areas (4.90 g kg^−1^), which was more than 100% greater. The interaction between Al and Fe oxides and OC is the main stabilising agent of OC (Matus et al., 2014; Shen et al., 2018). The impact of converting humic soils from their natural state to sugarcane cultivation on charcoal C and OC in Al/Fe-OM complexes in these soils in South Africa has not been investigated, despite their potential to attenuate climate change effects by increasing OC storage to deeper soil layers (> 2.0% C). To ensure sustainable agricultural yields and address climate change impacts, it is critical to comprehend the levels of C present in charcoal and Al/Fe-OM complexes across the soil profile, reaching a depth of 100 cm. The hypothesis was that a larger percentage of OC in humic soils is in the form of OC bound to Al/Fe-OM complexes and some in the form of charcoal C. The objective of the research was to assess the impact of land use and site conditions specifically, the comparison between sugarcane cultivation and forest on the levels of OC in charcoal C and Al/Fe-OM complexes in selected humic soils.

## Materials and methods

### Experimental sites

**Fig. 1:**
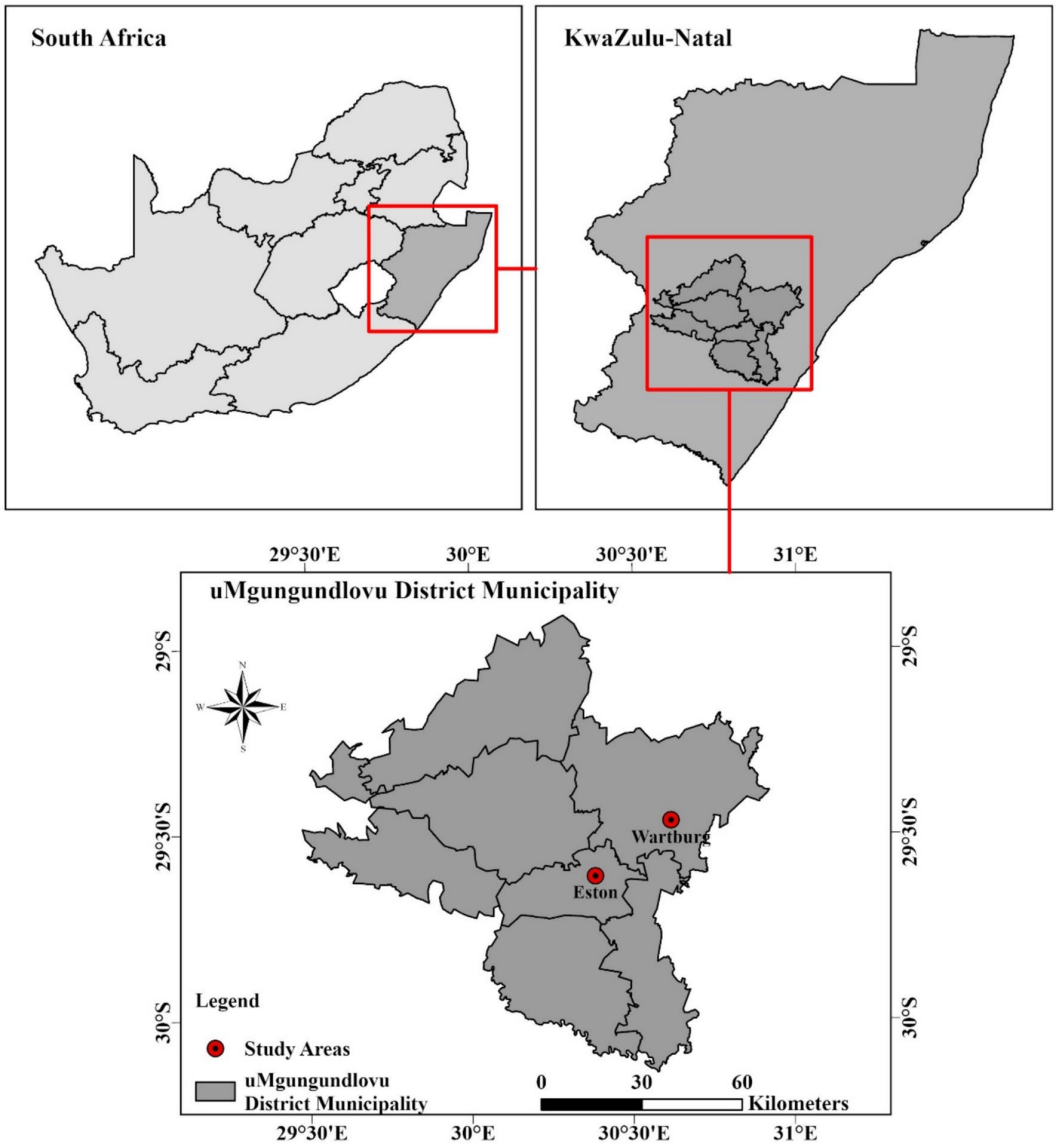
Location of study sites

The details of the study sites are based on farmers’ practices on each study farm and are among those used by Malepfane et al. (2022). The Eston (29°37′S; 30°23′E) and Wartburg (29°28′S; 30°37′E) sites had commercial wattle (*Paraserianthes lophanth*a) forests adjacent to the sugarcane (*Saccharum officinarum*) fields (Fig. 1). The Eston site had a minimum air temperature of 12.4 °C to a maximum air temperature of 24.2 °C with a mean annual precipitation of 805 mm and an elevation of 928 m. The Wartburg site had a minimum air temperature of 15.4 °C to a maximum of 26.7 °C, a mean annual precipitation of 798 mm, and an elevation of 933 m. Fertiliser nitrogen, phosphorus, and potassium (NPK) were applied regularly depending on soil fertility analysis results, and dolomitic lime was applied at a rate of 1 to 10 t ha^−1^ to reduce the soil acid saturation to 20% permissible acid saturation before sugarcane cultivation. The amount of dolomitic lime applied varied depending on soil acid saturation levels. Additionally, poultry manure was applied at a rate of 10 t ha^−1^ in the sugarcane field at the Eston and Wartburg sites. Before re-planting sugarcane, kale (*Brassica oleracea* var. *sabellica*) is usually planted as a rotation crop at the Wartburg site. No lime, fertiliser, or irrigation was applied to the wattle forests at either the Eston or Wartburg sites and litter accumulated on the soil surface. All sites practiced pre-harvest burning of sugarcane and removed fresh trash and residues from the soil surface. For each site, the soils cultivated with sugarcane were compared with the adjacent ones under forest management.

### Experimental design and soils

**Fig. 2:**
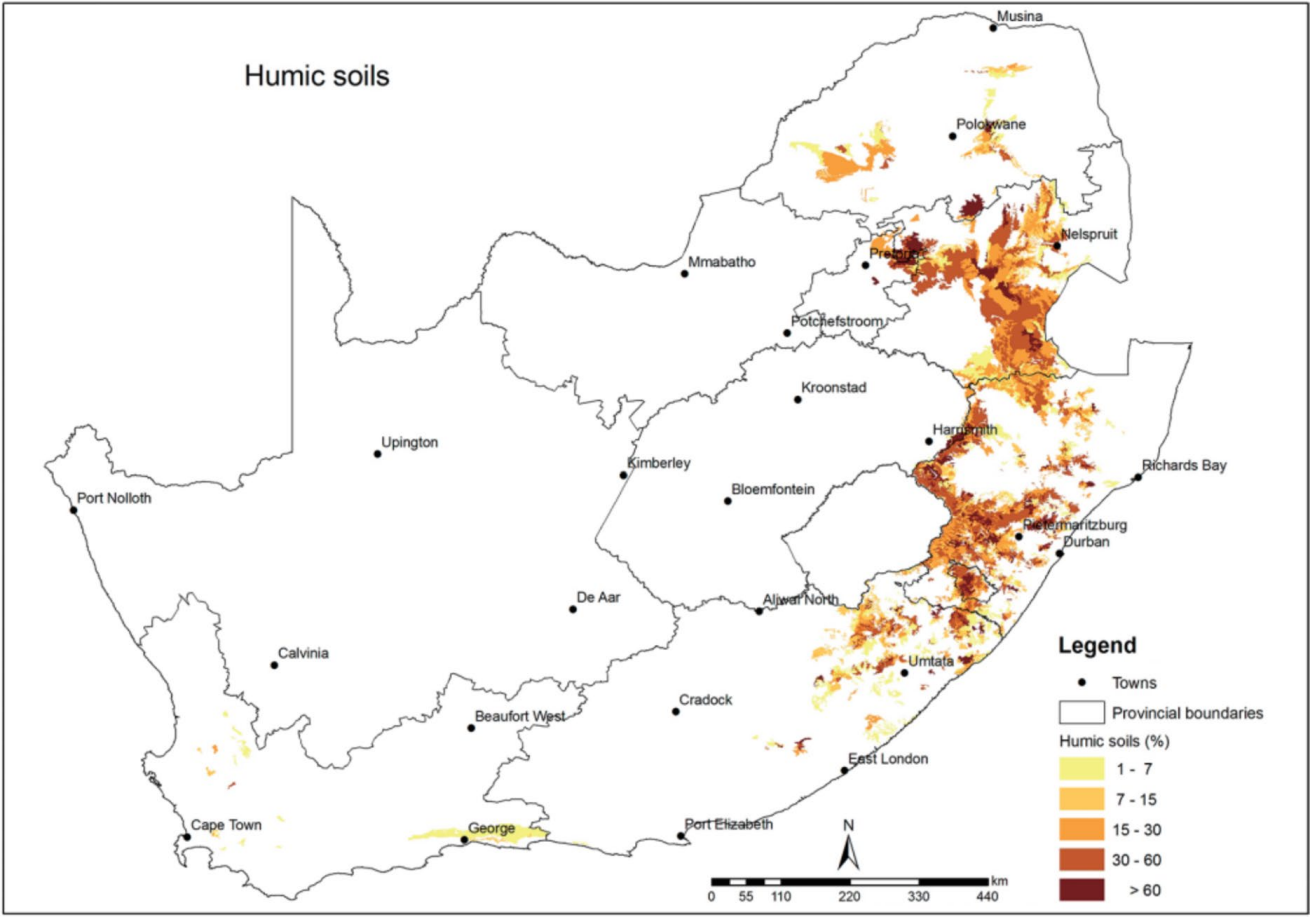
Humic soil distribution in South Africa (Fey, 2010)

The soils used in this study were clay loam from Wartburg and sandy clay loam from Eston. Three replicate fields with a size of approximately 0.1 to 0.3 ha were sampled for each land use per site. From each field, three subplots were randomly selected, and a pit was dug in each subplot. In each subplot, composite samples were collected at different depths with depth increments of 0–5, 5–10, 10–15, 15–20, 20–30, 30–40, 40–50, 50–60, 60–80, and 80–100 cm. The soil samples were air-dried, homogenised, and sieved (< 2 mm) before analysis. The soils from Eston, both sugarcane and forest, were classified locally as Magwa soil form with a thick humic A horizon overlying a yellow–brown apedal B horizon over unspecified material (Soil Classification Working Group, 2018), which translates to umbric xanthic Ferralsols according to the World Classification System (IUSS Working Group, 2014). The soils from the Wartburg site (sugarcane and forest) were classified locally as Inanda soil form with a thick humic A horizon overlying a red apedal B horizon over unspecified material (Soil Classification Working Group, 2018), translating to umbric rhodic Ferralsols according to the World Classification System (IUSS Working Group, 2014). All the soils used were derived from the Natal Group sandstone parent material (Soil Classification Working Group, 2018). The distribution of humic soils in South Africa is shown in Fig. 2. The soil pH of the study sites were below pH < 5.00 (Malepfane et al., 2022).

The total carbon content was determined using the LECO TruMac CNS Auto-analyser version 1.1x (Leco Corporation, 2012 St. Joseph, USA). The distribution of total carbon in the soil profiles were presented in Table 1. The average total carbon content was 22.2 and 29.8 g kg^−1^ for Eston sugarcane and forest, respectively, and for Wartburg, the total carbon was 19.4 and 37.6 g kg^−1^ in sugarcane and forest, respectively. Soil particle size distribution was determined using the pipette method as described by Gee and Bauder (1986) after removal of organic matter using hydrogen peroxide as described by van Reeuwijk, (1995). The samples were then dispersed with Calgon solution followed by sieving to separate the sand fraction, while the clay and silt content were determined through sedimentation. Thereafter, the textural class was determined using the textural triangle (Soil Classification Working Group, 1991). The clay content in the sugarcane and forest soils at Wartburg site was on average 37.6 and 43.8%, respectively. For the Eston site, sugarcane and forest soils had, on average, 33.9 and 24.3% clay content, respectively. The Fe and Al were extracted using the Mehlich 3 method (Mehlich, 1984). The concentration of Fe and Al in the samples was determined using the inductively coupled plasma atomic emission spectroscopy Varian 720-ES ICP-AES (Varian, California, USA). The acid saturation at the Eston site under forest was 80.6 and 83.8% in the 0–5- and 5–10-cm depths, respectively, while in the sugarcane soils, it was had 0.523 and 17.4% for the same depths, respectively.

**Table 1:** Soil organic carbon content (g kg^−1^) at different sites (Malepfane et al., 2022)

Depth	Eston		Wartburg	
cm	Sugarcane	Forest	Sugarcane	Forest
0–5	30.7abcd	63.1ef	27.2abcd	73.9 fg
5–10	29.5abcd	93.9 g	26.1abcd	52.2def
10–15	25.1abcd	28.5abcd	25.9abcd	42.9cde
15–20	23.6abcd	28.8abcd	22.8abc	39.7bcde
20–30	21.0abc	18.8abc	23.6abcd	34.2abcde
30–40	25.2abcd	20.6abc	19.7abc	33.4abcd
40–50	24.1abcd	16.2abc	17.9abc	29.7abcd
50–60	18.3abc	12.8ab	15.5abc	27.6abcd
60–80	13.3ab	9.04a	10.3a	23.7abcd
80–100	10.9ab	6.23a	4.88a	18.5abc
*p* value	0.007			
LSD	14.1			

Different lowercase letters indicate significant differences (*p* < 0.05) among treatment

### Charcoal C method validation

**Fig. 3:**
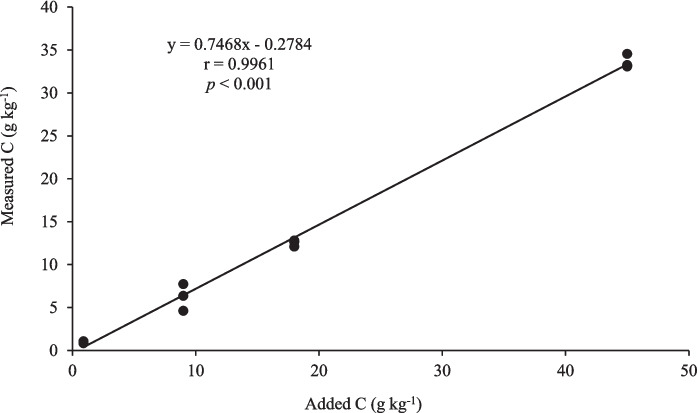
Charcoal C retained at different application rates of charcoal (pine bark biochar) after digestion

A preliminary experiment was conducted with the soil from the Eston (forest) site with less OC content from the 80–100 cm depth to determine the efficiency of the recovery of charcoal C using the method used in this study. Biochar (used as charcoal) was produced from pine bark (< 2-mm sieve size) from Cramnond Farm and pyrolysed at 650 °C in a furnace with no oxygen to avoid combustion. The biochar was sieved to < 500 µm to increase contact with the soil particles and applied to 20 g of soil to represent charcoal C at 0, 0.05, 0.1, 0.5, 1.0, 2.0, and 5.0% charcoal C (w/w). The amended samples were then digested with 30% hydrogen peroxide (H_2_O_2_) and 1 M nitric acid (HNO_3_) on a hot plate for 16 h, as described by Kurth et al. (2006), and the total C remaining in the sample was analysed using LECO TruMac CNS Auto-analyser version 1.1x (Leco Corporation, 2012 St. Joseph, USA). The results were used to check the effectiveness of the method. For this, the linear regression model was used to determine the relationship between added charcoal C and measured charcoal C using Microsoft Excel software, as indicated in Fig. 3. From the results, the added charcoal C explained > 99% of the variation of the measured charcoal C, indicating the efficiency of the method. The method was then used for further analysis of charcoal C in the different soil samples.

### Determination of charcoal C content and stocks

The samples were analysed for charcoal C concentration using the digestion method as described by Kurth et al. (2006), with two replicates for each depth. For this, a 1-g dried sample (< 500 µm) in a 250-mL Erlenmeyer flask was treated with 20 mL of 30% hydrogen peroxide (H_2_O_2_) and 10 mL of 1 M nitric acid (HNO_3_). The flask was swirled while mixing at room temperature for over 30 min under the fume hood. Thereafter, the flask was heated using a heating plate at 100 °C for 16 h, with occasional swirling to observe the occurrence of effervescence. After 16 h, the flask that showed effervescence was heated for another 4 h on the heating plate. Thereafter, the samples were filtered into storage bottles through Whatman filter paper No. 1, and the residues on the filter paper were oven-dried at 60 °C and ground using a mortar and pestle. The total C remaining in the sample, which was considered charcoal C, was then determined by dry combustion with the LECO TruMac CNS Auto-analyser version 1.1x (Leco Corporation, 2012 St. Joseph, USA). The total C in the soil samples from all the sites was determined using the LECO TruMac CNS Auto-analyser version 1.1x (Leco Corporation, 2012 St. Joseph, USA). The charcoal C stock was determined using the following equation (Malepfane et al., 2022):

1 $$Charcoal\;C\;stocks\;\left(Mg\;C\;ha^{-1}\right)=C\times BD\times profile\;depth\times10,000\times0.001$$

where C is charcoal C (g kg^−1^); BD is bulk density (g m^−3^); profile depth is the layer depth (m); 10,000 is a conversion factor for m^−2^ to ha^−1^, and 0.001 is a conversion factor for kg to Mg.

### Determination of organic carbon, aluminium (Alp), and iron (Fep) in Al/Fe-OM complexes

The OC, Al, and Fe associated with Al/Fe-OM complexes were extracted with Na-pyrophosphate as described by Blakemore (1981). For this, 50 mL of 0.1 M Na-pyrophosphate was added to a 1-g soil sample and shaken for 16 h using a reciprocal shaker at 180 r.p.m. Thereafter, five drops of 0.4% superfloc were added while shaking vigorously before centrifugation at a speed of 20,000 r.p.m (39,872 × *g*) for 30 min. The supernatant was then filtered through Whatman No. 1 filter paper, and 1 mL of each extract was diluted 20 times with deionised water. The concentration of Al and Fe in the Na-pyrophosphate extract (Alp and Fep) was determined using an inductively coupled plasma atomic emission spectrometry Varian 720-ES ICP-AES (Varian, California, USA). In the same extract, the OC associated with Al/Fe bound OC was analysed using the Walkley–Black method as described by Garrido and Matus (2012).

### Statistically data analysis

The data for charcoal C and stocks, total C, Al/Fe bound OC, Alp, and Fep were subjected to a general analysis of variance using GenStat Ed. 18 (Boer et al., 2015) comparing land use, site, and depth. The least significant difference (LSD), at a 5% level, was used to compare treatment means (land use, site, and depth). The fisher’s protected least significant test was applied to separate treatment means at *p* < 0.05. Analysis of the correlation between charcoal C and measured total OC were determined using GenStat Ed. 18 (Boer et al., 2015) and the correlation coefficient and *p*-value of the relationship recorded. A principal component analysis (PCA) was applied separately to show the overall effect of land use on different soil parameters in the R Statistical Software 2018 (R Core Team, 2018).

## Results

### Charcoal C concentration and stocks, and Al/Fe-bound OC

**Fig. 4:**
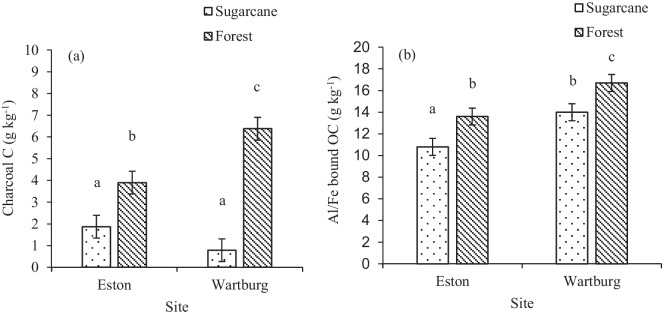
Effects of land use on charcoal C and Al/Fe bound OC concentration. Different lowercase letters indicate significant differences (*p* < 0.05) among treatment. **a** and **b** indicate charcoal C and Al/Fe-bound OC, respectively (*n* = 120). The error bars indicate standard error of means

Land use had a significant effect on charcoal C concentration (Fig. 4a). Eston and Wartburg sugarcane had statistically similar charcoal C concentration, which was significantly lower than the forest land use at both sites. Wartburg forest had significantly higher (63.6% higher) charcoal C compared to Eston forest. In the Wartburg site, sugarcane cultivation significantly reduced charcoal C concentrations (0.794 g kg^−1^) when compared to the uncultivated forest (6.38 g kg^−1^). At Eston, charcoal C in sugarcane (22.2 g kg^−1^) and forest (29.8 g kg^−1^) soils constituted 8.42 and 13.1% of the total soil OC, respectively. For the Wartburg site, sugarcane and forest land use constituted 4.07 and 17.0% of the total OC, respectively. Aluminium/Fe-bound OC was significantly affected by land use (Fig. 4b). Wartburg forest had significantly higher Al/Fe-bound OC compared to sugarcane by 19.3% (Fig. 4b). Eston sugarcane had significantly lower Al/Fe-bound OC compared to both sugarcane and forest at Wartburg and Eston forest. Sugarcane cultivation reduced Al/Fe-bound OC by 25.9% compared to forest land use in Eston. The Al/Fe-bound OC in Eston and Wartburg under sugarcane constituted 48.7 and 72.2% of the total OC, respectively. The Al/Fe-bound OC in the Eston and Wartburg sites under forest constituted 45.6 and 44.5% of the total OC, respectively. The interaction effect between land use, site, and depth on charcoal C distribution was significant (Table 2). Significantly higher charcoal C was observed in the Wartburg forest in the 5–10-cm depth (18.3 g kg^−1^) compared to all the other depths and land use. The Wartburg forest at 0–5-cm depth was similar to the Eston forest at 5–10-cm depth and significantly higher than the other depths except for the Wartburg forest 15–20-cm depth and Eston forest at 0–5- and 20–30-cm depths (Table 2). For both sites, the sugarcane land use had significantly lower charcoal C except for 0–10-cm depth at Eston.

**Table 2:** Distribution of charcoal C (g kg^−1^) within the soil profile in sugarcane and forest land use

Depth	Eston		Wartburg	
cm	Sugarcane	Forest	Sugarcane	Forest
0–5	2.82abc	5.88bcd	2.93abc	12.4e
5–10	10.0de	4.00abc	1.58abc	18.3f
10–15	2.95abc	3.53abc	0.99a	3.93abc
15–20	1.70abc	4.20abc	0.65a	6.28 cd
20–30	ND	6.16 cd	1.23ab	4.64abc
30–40	0.19a	3.66abc	ND	4.50abc
40–50	0.84a	4.14abc	0.19a	3.35abc
50–60	0.13a	2.34abc	ND	4.27abc
60–80	0.03a	3.37abc	0.24a	2.98abc
80–100	ND	1.75abc	0.11a	3.16abc
*p* value	0.021			
LSD	4.73			

Different lowercase letters indicate significant differences (*p* < 0.05) among treatment. ND indicates below detection

Higher Al/Fe-bound OC was observed in the forest land use at the top 0–5- and 5–10-cm depths, compared to all other depths for both land uses, except for the 10–15-cm depth under forest, which was similar to the 5–10-cm depth (Table 3). The concentration of Al/Fe-bound OC in the top 10-cm depth of the forest land use was above 2.00%. For the sugarcane land use, the top 50-cm depth had a similar concentration of Al/Fe-bound OC compared to the deeper layers (Table 3). Overall, the Al/Fe-bound OC decreases with an increase in soil depth for both land uses. However, there was still evidence of Al/Fe-bound OC in the deeper layers. The interaction effect between site, land use, and depth on Al/Fe-bound OC was not significant (results not shown); only the effect of land use and depth on Al/Fe-bound OC was significant (Table 3). The interaction between site, land use, and depth did not affect charcoal C stocks (results not shown); only land use and depth significantly affected the charcoal C stocks (Table 4). Sugarcane demonstrated a significant decrease in charcoal C stocks with depth, while forest land use showed no change in charcoal C stocks with depth. From 20–30-cm depth, the charcoal C stocks in forest land use were significantly higher than each corresponding depth under sugarcane land use. The concentration of Fep was significantly affected by the interaction between site, land use, and depth (Table 5). For Eston, the sugarcane land use at 0–5- and 5–10-cm depth were significantly lower than the 30–40- to 80–100-cm depths. Whereas for the forest, only the 80–100-cm depth had a significantly lower Fep concentration.

**Table 3:** Concentration of OC bound in Al- and Fe-OM complexes (g kg^−1^) under different land use and depths

Depth (cm)	Sugarcane	Forest
0–5	16.1def	25.8 h
5–10	15.7def	23.0gh
10–15	15.4def	19.9 fg
15–20	11.9bcd	16.1def
20–30	11.9bcd	13.3cde
30–40	13.3cde	17.1ef
40–50	12.6cde	9.77abc
50–60	11.2abcd	11.2abcd
60–80	9.07abc	9.07abc
80–100	6.98ab	6.63a
*p* value	0.026	
LSD	5.01	

Different lowercase letters indicate significant differences (*p* < 0.05) among treatment. OC, OM, Al, and Fe represent organic carbon, organic matter, aluminium and iron

**Table 4:** Charcoal C stocks (Mg C ha^−1^) distribution in sugarcane and forest land use within the soil profile

Depth (cm)	Sugarcane	Forest
0–5	1.89abcde	3.38bcde
5–10	3.54bcdef	5.65 fg
10–15	1.14abcd	2.13abcde
15–20	0.741abc	2.97abcdef
20–30	0.752abc	7.87 g
30–40	0.115a	4.99efg
40–50	0.735abc	3.72cdef
50–60	0.076a	4.17def
60–80	0.334ab	7.88 g
80–100	0.110a	6.11 fg
*p* value	0.045	
LSD	3.23	

Different lowercase letters indicate significant differences (*p* < 0.05) among treatment

**Table 5:** Distribution of Fep (g kg^−1^) within the soil profile in sugarcane and forest land use

Depth	Eston		Wartburg	
cm	Sugarcane	Forest	Sugarcane	Forest
0–5	2.65a–f	3.76a–j	2.11a–d	1.38ab
5–10	2.53a–e	6.05d–j	2.22a–d	2.18a–d
10–15	3.81a–j	7.14 h–j	4.47b–j	5.12b–j
15–20	5.52c–j	6.06d–j	5.49c–j	7.36ij
20–30	4.73b–j	5.33b–j	3.07a–g	4.00b–j
30–40	6.97 g–j	6.64f–j	4.61b–j	4.45b–j
40–50	6.67 g–j	7.23 h–j	4.59b–j	3.46a–j
50–60	6.80 g–j	6.95 g–j	3.30a–h	7.30ij
60–80	7.57j	4.88b–j	6.44e–j	7.59j
80–100	7.15 h–j	1.81a–c	ND	6.00d–j
*p* value	0.0.024			
LSD	2.91			

Different lowercase letters indicate significant differences (*p* < 0.05) among treatment. ND indicates below detection. Fep indicate iron bound to organic matter

### Alp and Fep in Al/Fe-organic matter complexes

**Fig. 5:**
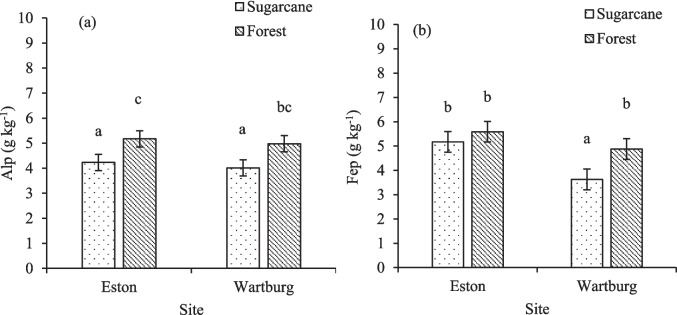
Effects of land use on Alp and Fep concentration. Different lowercase letters indicate significant differences (*p* < 0.05) among treatment. **a** and **b** indicate Alp and Fep concentrations, respectively (*n* = 120). The error bars indicate standard error of means

**Fig. 6:**
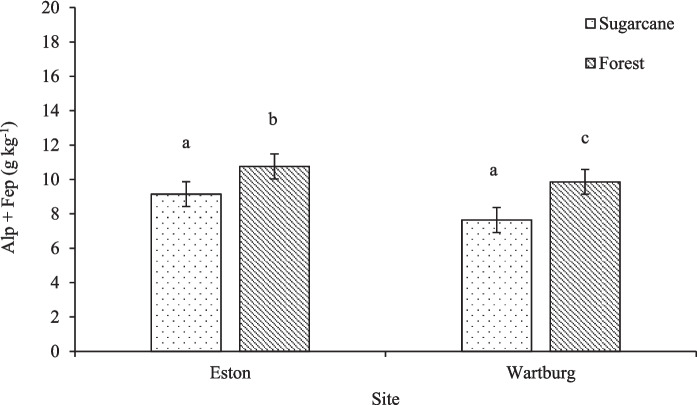
Effects of land use on Alp + Fep concentration. Different lowercase letters indicate significant differences (*p* < 0.05) among treatment (*n* = 120). The errors bar indicate standard error of means

The Alp showed a significant response to land use but not to the interaction between land use and depth (Fig. 5a). Higher Alp concentration was observed in Eston and Wartburg forests when compared to Wartburg sugarcane. Sugarcane cultivation in Wartburg significantly reduced Alp concentration by 24.2% compared to the forest and in Eston sugarcane cultivation reduced charcoal by 22.2%. For the Fep, land use and depth had a significant effect, but not the interaction between depth and land use (Fig. 5b). The Fep was significantly lower in Wartburg sugarcane compared to the other land use in both Wartburg and Eston. Sugarcane cultivation reduced Fep by 34.4% compared to the forest in Wartburg (Fig. 5b). In Eston site, both sugarcane and forest had similar Fep concentrations. Land use in the Eston site did not affect the Alp + Fep (Fig. 6). At Wartburg, sugarcane cultivation reduced Alp + Fep by 29.1% when compared to forest land use. Compared to Eston, the concentration of Alp + Fep in the Wartburg forest was similar to that of sugarcane and forest.

### Principal component analyses of management effects on different soil parameters

**Fig. 7:**
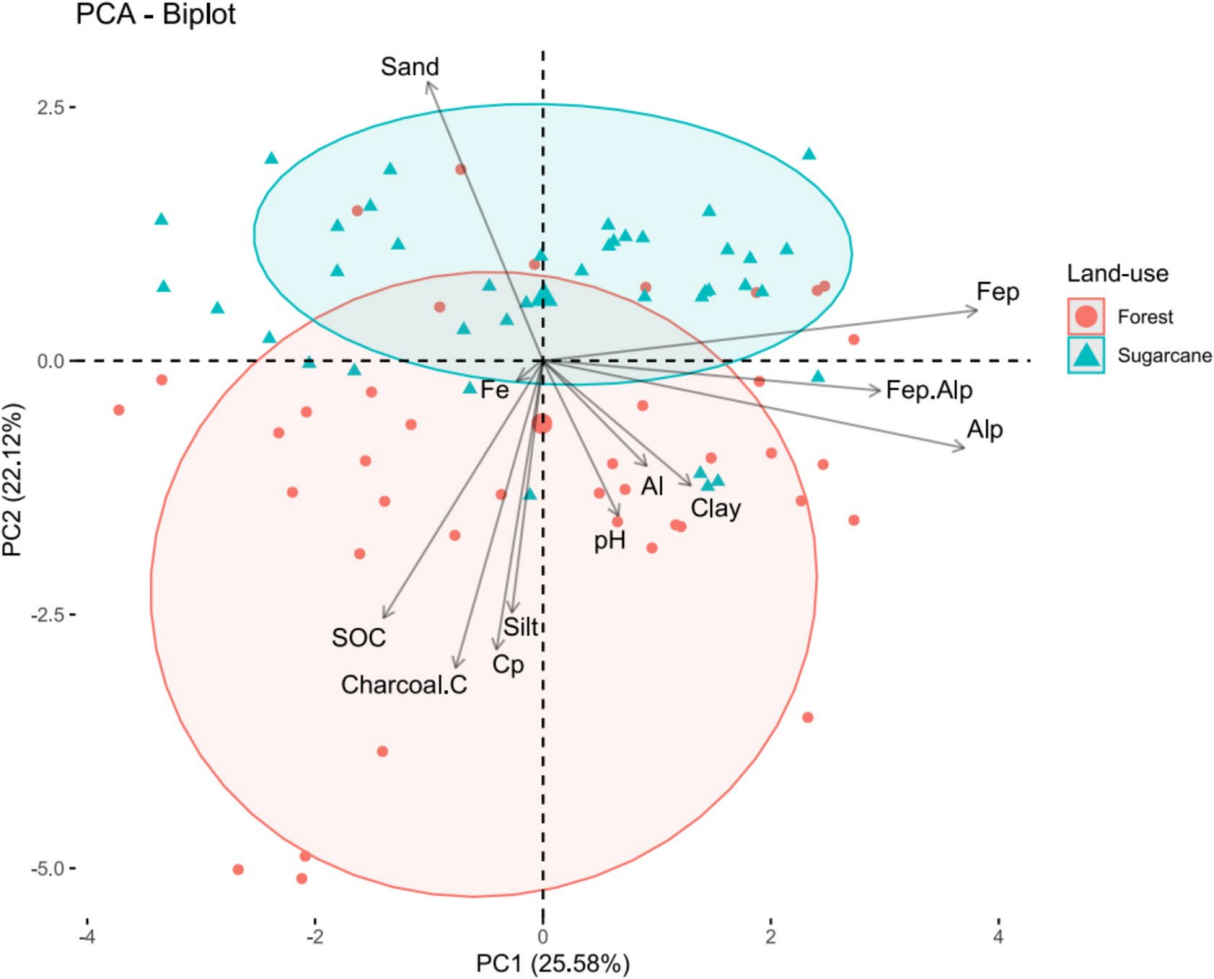
The first two axes of principle component analysis are based on effect of land use on different soil parameters. The axes explained 47.7% variation using PC1 and PC2. Groups represent the different managements where forestry (uncultivated) sugarcane (cultivated) land use

The principal component analysis (PCA) showed that the first three PCAs accounted for 65.3% of the variability within the dataset, with the first two PC explaining 47.7% of the variation (Fig. 7). The first and second PC explained 25.58 and 22.12% of the variability of the data, respectively. Charcoal C, Al/Fe-bound OC (Cp), SOC, and silt, and all these parameters were negatively correlated to sand. The organically bound Fep, Alp, and Fep + Alp were positively correlated. The sand was negatively correlated to soil pH, Mehlich 3 extractable Al, and clay. The forest land use was characterised by high charcoal C, SOC, Al/Fe-bound OC, silt, clay, and Mehlich 3 extractable Fe and Al, while the soils under sugarcane cultivation were characterised by high sand, organically bound Fep, Fep + Alp, and Alp, and lower clay content.

## Discussion

### Distribution of charcoal C and its stocks

The addition of incomplete burnt biomass, which produces charcoal C, could explain the higher charcoal C content in the Wartburg and Eston forest sites compared to sugarcane cultivation (Wang et al., 2017). Burning biomass converts soil biomass C to charcoal C, which returns to the soil over time and transforms from a labile C to a passive charcoal C fraction (Boot et al., 2015). In addition, limited soil disturbance in forest soils could limit the decomposition of charcoal C over time, hence increasing charcoal C stocks. The higher amount of charcoal C in the Wartburg forest site (6.38 g kg^−1^) might also be due to the higher amount of clay content, which provides better physical and chemical protection for the charcoal C as part of the POM-C aggregate. In addition to biomass burning, the historical practice of slash and burn cultivation in the Wartburg forest may have contributed to the distribution of charcoal C within the soil profile (Tomson et al., 2021). Management strategies that favour the translocation of charcoal C could also explain the evidence of charcoal C at subsoil layers. Moreover, the high water conservation capacity of forest soils in general, which enables the movement of soil particles down the soil profile, especially in these well-drained soils (Fey, 2010), could explain the high charcoal C content in forest land use in Eston and Wartburg sites.

The high acid saturation levels (> 80%) in Eston forest limit microbial decomposition in soils (Malik et al., 2018), thereby reducing overall charcoal C losses and increasing its concentration in soils over time. The lower concentration of charcoal C in the soil under sugarcane at the Wartburg and Eston sites could be attributed to the addition of lime, and organic and inorganic fertilisers. These fertilisers increase the activity of microorganisms, leading to the rapid decomposition of resident soil C, including the charcoal C fraction. Knicker (2011) reported that there is evidence that charcoal C is not as recalcitrant as commonly assumed, and this can explain the decrease in its concentration in the soil under sugarcane in both sites. A study conducted by Hilscher and Knicker (2011) showed that pyrogenic soil organic matter derived from grass biomass can be decomposed by microorganisms, although the process occurs at a slow rate (Boot et al., 2015; Krishnaraj et al., 2016). Additionally, the lower charcoal C in sugarcane for both sites could be explained by rapid decomposition due to the addition of labile poultry litter manure, which could increase microbial activities, hence increasing the overall decomposition of OC, including the charcoal C pool. The lower charcoal C under sugarcane in Wartburg and Eston sites could also be due to more frequent burning as the sugarcane is under pre-harvest burning management, resulting in greater combustion of charcoal C and losses as a result of runoff and leaching/eluviation. Muqaddas et al. (2015) reported a 44% decrease in charcoal C after 2 years of continuous burning, which was consistent with this observation. The accumulation of charcoal C in deeper layers in humic soils, which are porous and have greater drainage (Fey, 2010), confirms the potential for leaching or eluviation and could facilitate its movement down the soil profile. In addition, the deep roots in both sugarcane and forest land use could explain the accumulation of charcoal C in deeper soil layers. The decrease in charcoal C with depth was also reported in several articles in the literature (Boot et al., 2015; Hobley et al., 2017; Huang et al., 2018; Pingree et al., 2012; Wang et al., 2017; Zhan et al., 2013; Zhao et al., 2016).

### Effects of land use on Al/Fe-bound OC and Alp- and Fep-organic complexes

Soils under forest in Wartburg and Eston had a higher concentration of OC bound to Fe/Al bound-OM complexes than those in sugarcane land use. This could be explained by the high OC content in forest soil in these sites, where a major portion of it could be bound to Al/Fe-OM complexes, and with limited microbial activities due to the high level of acidity, the OC will accumulate for longer. More importantly, high soil acidity and low soil pH (4.2–5.0) result in high mobility of Al and Fe in soil solutions, which are freely available to be bound with OC in soil, resulting in more stabilisation of the OC over time in these soils (Matus et al., 2014; Parada et al., 2024). In soils with low soil acid saturation levels, as a result of liming, which is the case for sugarcane land use at both sites, the solubility of Al and Fe is reduced, decreasing their ability to bind with OC (Parada et al., 2024). As a result, the OC would be available for microbial degradation, resulting in a decrease in overall OC bound to Al and Fe over time. After 43 years, a long-term study in Andisol found that land use and liming reduced Al/Fe-OM complexes by 36–40% when compared to the native forest in the top 20-cm depth (Parada et al., 2024). Jamoteau et al. (2024) reported that mineral-organic associations are essential for OC reserves but then cultivation disrupts these associations resulting in losses of OC over time. The effect of Al and Fe on OC stabilisation was further evidenced by the high Mehlich 3 Al and Fe in forest soils when compared to sugarcane land use (Fig. 7). Malepfane et al. (2022) suggested that the high concentration of Mehlich 3 extractable Al and Fe in humic soils could explain this. The OC bound to Al/Fe-OM complexes was more than 40% of the total soil C in all the studied soils, which was similar to Zhao et al. (2016), who observed that on average, Fe-bound OC only contributed 37.8% to the total C in soil. A recent study observed less that OC bound to Al/Fe in volcanic soils of Japan accounted for 2.3 to 15.1% (8.4% on average) of the OC content and was found to be the most OC stabilisation mechanism (Shindo & Inoue, 2024). Basile-Doelsch et al. (2009) demonstrated that between 58 and 80% of the organic matter in Ferralsols was stabilised through organo-mineral complexation depending on the horizon. Deep roots in both forest and sugarcane land use could explain the evidence of Al/Fe-bound OC at deeper soil layers. In addition, the evidence of Al/Fe-bound OC at deeper soil layers could be explained by the effects of cultivation in sugarcane land use, which could have resulted in vertical migration of organo-mineral complexes and accumulation in deeper soil depths (Basile-Doelsch et al., 2009). These roots could produce organic acid that chelates with Al and Fe in soil solution, thereby increasing OC accumulation in deeper soil layers. Also, Malepfane et al. (2022) showed the evidence of Al and Fe in deeper soil layers, which could bound with OC and accumulate in the deeper soil layer. The higher Al and Fe concentrations in these soils could have been extracted, resulting in high concentrations of Al and Fe in the form of Alp and Fep, masking the differences within management, especially for the Eston site.

## Conclusion

Charcoal C makes up a small proportion of total C in the studied humic soils (Ferralsols), in both sugarcane and forest land use, and the concentration and stocks are reduced by sugarcane cultivation in both sites. Similar to charcoal C, land use change from wattle forest to sugarcane cultivation in both sites reduced the concentration of the Al/Fe-bound OC. The high C in humic soils is stabilised by Fe and Al oxides as Fe-organic matter and Al-organic matter, hence increasing the concentration of protected C. These findings demonstrate that Fe/Al-organic matter complexes are more important than charcoal C fraction as a C stabilisation mechanism, and this explains the high C in these soils. These findings imply that organo-mineral complexes can be used to estimate the carbon sequestration capacity of these humic soils. However, these results also suggest that, while the OC in humic soils is high, land use change from forest to sugarcane cultivation destabilises the Al/Fe-OM complexes and charcoal C in these soils. While the study was only conducted in two land uses (wattle forest and sugarcane) under similar conditions, the applicability of the findings under other land uses and different climatic conditions are not guaranteed. Therefore, future work should focus on the processes involved in OC stabilisation and degradation in humic soils under different climatic conditions with other land uses.

## Data Availability

No datasets were generated or analysed during the current study.
